# Electrochemical Hydrogen Evolution over Hydrothermally Synthesized Re-Doped MoS_2_ Flower-Like Microspheres

**DOI:** 10.3390/molecules24244631

**Published:** 2019-12-17

**Authors:** Juan Aliaga, Pablo Vera, Juan Araya, Luis Ballesteros, Julio Urzúa, Mario Farías, Francisco Paraguay-Delgado, Gabriel Alonso-Núñez, Guillermo González, Eglantina Benavente

**Affiliations:** 1 Departamento de Química, Universidad Tecnológica Metropolitana, Las Palmeras 3360, Ñuñoa, Santiago, Chile; p.vera1@gmail.com; 2 Centro de Investigaciones Costeras de la Universidad de Atacama (CIC-UDA), Universidad de Atacama, Copayapu 485, Copiapó, Chile; juan.araya@uda.cl; 3 Instituto de Ciencias Químicas Aplicadas, Universidad Autónoma de Chile, El Llano Subercaseaux 2801, San Miguel, Chile; luis.ballesteros@uautonoma.cl; 4 Departamento de Ciencias Farmacéuticas, Facultad de Ciencias, Universidad Católica del Norte, Casilla 1280, Antofagasta, Chile; j.urzua.ahumada@gmail.com; 5 Centro de Nanociencia y Nanotecnología, Universidad Nacional Autónoma de México, Ensenada C. P. 22860, Mexico; mario@cnyn.unam.mx (M.F.); galonso@cnyn.unam.mx (G.A.-N.); 6 Departamento de Física de Materiales, Centro de Investigación Materiales Avanzados S.C., Miguel de Cervantes 120, CP 31136, Chihuahua, Mexico; francisco.paraguay@cimav.edu.mx; 7 Departamento de Química, Facultad de Ciencias, Universidad de Chile, Las Palmeras 3425, Santiago, Chile; ggonzale@uchile.cl

**Keywords:** molybdenum disulfide, rhenium doping, hydrothermal synthesis, HER, hydrogen evolution reaction

## Abstract

In this research, we report a simple hydrothermal synthesis to prepare rhenium (Re)- doped MoS_2_ flower-like microspheres and the tuning of their structural, electronic, and electrocatalytic properties by modulating the insertion of Re. The obtained compounds were characterized by X-ray diffraction (XRD), scanning electron microscopy (SEM), high-resolution transmission electron microscopy (HRTEM), Raman spectroscopy, and X-ray photoelectron spectroscopy (XPS). Structural, morphological, and chemical analyses confirmed the synthesis of poorly crystalline Re-doped MoS_2_ flower-like microspheres composed of few stacked layers. They exhibit enhanced hydrogen evolution reaction (HER) performance with low overpotential of 210 mV at current density of 10 mA/cm^2^, with a small Tafel slope of 78 mV/dec. The enhanced catalytic HER performance can be ascribed to activation of MoS_2_ basal planes and by reduction in charge transfer resistance during HER upon doping.

## 1. Introduction

The use of hydrogen (H_2_) as fuel has gained significant importance. Hydrogen is a source of clean energy obtained at convenient cost by the water electrolysis process [1,2]. This process has proven to be one of the most efficient methods for hydrogen production; however, the use of high-cost and scarce precious metal (Pt, Pd) materials, which have excellent electrocatalytic performances, hinders their large-scale application. In this context, several earth-abundant catalytic alternatives have been investigated, including, for example, phosphide-based materials [3], transition metal monopnictides [4], metal carbides [5], and transition metal dichalcogenides [6], among others. Molybdenum disulfide (MoS_2_), one of the most earth-abundant transition metal dichalcogenides, has been extensively researched as a low-cost electrocatalyst for hydrogen evolution reaction (HER) [2,7]. Theoretical and experimental studies have demonstrated that the edges of the semiconducting 2H-MoS_2_ are the catalytically active sites toward HER, while the basal planes are inert [8,9]. Most strategies to improve HER performance of MoS_2_ electrocatalysts are consequently focused on phase, defects, and heterostructure engineering to maximally expose edge sites and to activate the basal plane [7,10,11]. Doping with non-metallic or transition metals atoms into the MoS_2_ structure activates both edges and basal plane, improving the electronic mobility, charge transportability, and catalytically active surface area, therefore enhancing HER activity of the material [12,13,14,15]. In this sense, rhenium doping has been proposed to tune the electronic structure and polymorphic phases and to activate the basal planes of MoS_2_ [16,17,18,19]. It has been demonstrated that a low concentration Re (n-type) doping induces broader valence bands and electron accumulation close to the Fermi level in MoS_2_ fullerenes, resulting in a better HER performance [20].

This work studies the influence of rhenium incorporation under hydrothermal conditions in the electrocatalytic behavior of Re-doped MoS_2_ for HER in acidic media. The Re-doped MoS_2_ samples were prepared by a direct in situ sulfurization of ammonium molybdate and ammonium perrhenate by thiourea, using a simple hydrothermal process. A pristine MoS_2_ material for direct comparison was also synthesized by the same method. Herein, we show morphological, structural, electronic, and electrocatalytic effects of a hydrothermal Re doping on MoS_2_, where the HER performance is tuned by the amount of Re doping.

## 2. Results

### 2.1. Characterization of the Catalyst

#### 2.1.1. Scanning Electron Microscopy Analysis

**Figure 1:**
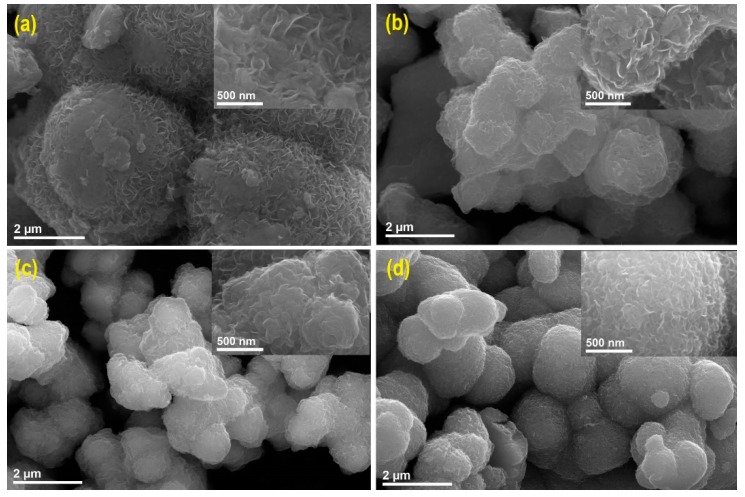
(**a**) SEM micrographs of pristine MoS_2_, (**b**) 14.7% Re-doped MoS_2_, (**c**) 27.7% Re-doped MoS_2_, and (**d**) 39.2% Re-doped MoS_2_ samples.

SEM measurements were carried out to characterize the morphology of all samples. SEM images of all samples showed an overall flower-like similar morphology, both in the undoped and Re-doped MoS_2_ samples (Figure 1).

These flower-like particles are composed of disordered nanosheets, which project their edges perpendicularly from the surface of a hierarchical structure. For the pristine MoS_2_ sample (Figure 1a), an agglomeration of sphere-like particles with sizes ranging from 1.0 to 3.0 µm is observed. With increasing Re content in the samples, a decreasing size of the MoS_2_ hierarchical structures is observed, with no perceptible changes in their morphology (Figure 1a–c). As it can been seen in Figure 1c,d, more discrete particles are observed for samples with higher rhenium content, with an average diameter of 1.63 μm for the 39.2% Re-doped MoS_2_ sample (Figure 1d). Higher magnification of the nanosheets reveals the Re-doped samples to have a smaller lateral size than that of the undoped sample, with the smallest size for the 39.2% Re-MoS_2_ sample (Insets Figure 1a–d). Similar trends have been observed for solvothermal MoS_2_, and MoSe_2_ materials doped with Cu, Ru, and V [14,21,22].

#### 2.1.2. X-ray Diffraction Analysis 

**Figure 2:**
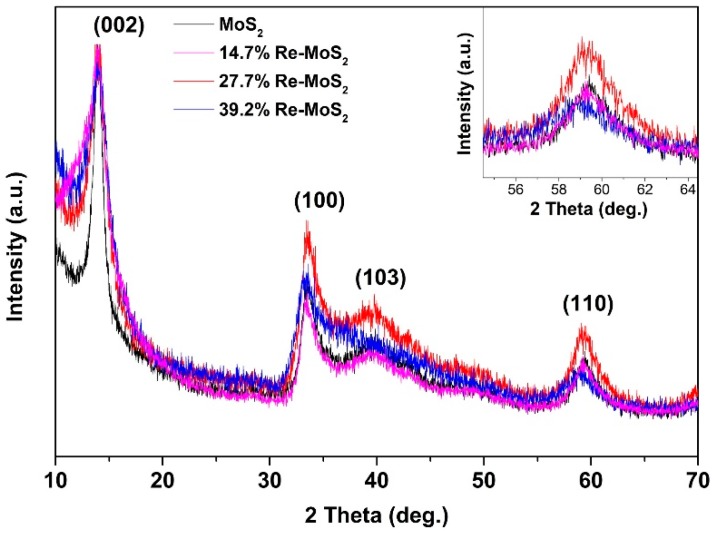
X-ray diffraction patterns of pristine MoS_2_, 14.7% Re-doped MoS_2_, 27.7% Re-doped MoS_2_, and 39.2% Re-doped MoS_2_ samples. Inset shows the (110) peak of all samples.

XRD was utilized to analyze the structural features of rhenium doping in all samples. As observed in Figure 2, the XRD profiles of pristine and doped samples look very similar, showing a single-phase with four reflections at approximately 2 θ = 14.3, 33.2, 39.5, and 58.7° corresponding, respectively, to (002), (100), (103), and (110) planes of 2H-phase MoS_2_ polytype (JCPDS 37–1492) [19,23]. Dominance of 2H-MoS_2_ diffraction peaks makes it difficult to determine the presence of the 1T_d_-ReS_2_ phase (which has a particularly disordered lattice structure) in all XRD sample patterns [23]. Although the Re-doped MoS_2_ and pristine MoS_2_ XRD profiles shown in Figure 2 look very similar, even with increasing rhenium content, they reveal a broadening of the (002) peaks and a gradual decrease of the I(002)/I(100) diffraction peaks ratio (2.2, 1.9, 1.3 for pristine MoS_2_, 14.7% Re–MoS_2_, and 27.7% Re–MoS_2_, respectively). This result indicates a concomitant decrease of the aligned (002) planes with increasing rhenium content in the samples, which is in agreement with similar MoS_2_ materials with disordered structures [24]. Sample 39.2% Re–MoS_2_, which presents the highest proportion of rhenium, shows the most amorphous structure, with a broad bulge in the 33–45° range, associated with merging of (100) and (103) planes, and by the presence of a low-intensity and shifted (110) peak (Inset Figure 2) [25].

#### 2.1.3. Raman Spectroscopy Analysis

**Figure 3:**
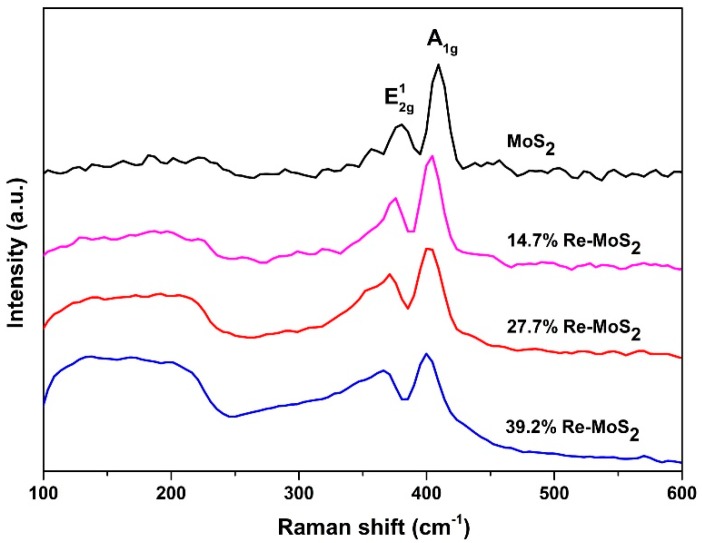
Raman spectra of pristine MoS_2_, 14.7% Re-doped MoS_2_, 27.7% Re-doped MoS_2_, and 39.2% Re-doped MoS_2_ samples.

Raman spectroscopy was utilized to characterize crystal phase and structural features of Re-doped MoS_2_ samples. As shown in Figure 3, Raman spectra of all samples display the typical two main lines of 2H-MoS_2_, corresponding to out-of-plane A_1g_ mode, and an in-plane E^1^_2g_ mode, observed at approximately 409–400 cm^−1^ and 382–371 cm^−1^, respectively [23,26]. By the increase of rhenium content, a remarkable line broadening is observed in the first-order Raman signals. The disordered layered arrangement in the Re-doped MoS_2_ samples agrees with the broadening of the lines and with the aspect ratio intensity of these peaks (A_1g_:E^1^_2g_) in all samples [26,27]. This has also been correlated with incorporating substitutional Re into the MoS_2_ structure [23,27]. Additionally, the intensity of the broadened band in the region between 100 and 250 cm^−1^ can also be attributed to Re content in the samples [28]. In this sense, the rhenium content could affect this Raman region through formation of a ReS_2_ single phase (ReS_2_ Raman active strongest vibrations are located in the range of 120 to 240 cm^−1^) [29,30] by inducing changes in the MoS_2_ phase (from 2H to 1T/1T_d_) [16,18,31] or by defect-induced scattering of the MoS_2_ small/disordered particles (low-frequency defect-activated modes) [26,28,32]. The overlapping frequencies of these variables preclude a particular identification for the origin of these bands.

#### 2.1.4. Scanning Transmission Electron Microscopy Analysis

**Figure 4:**
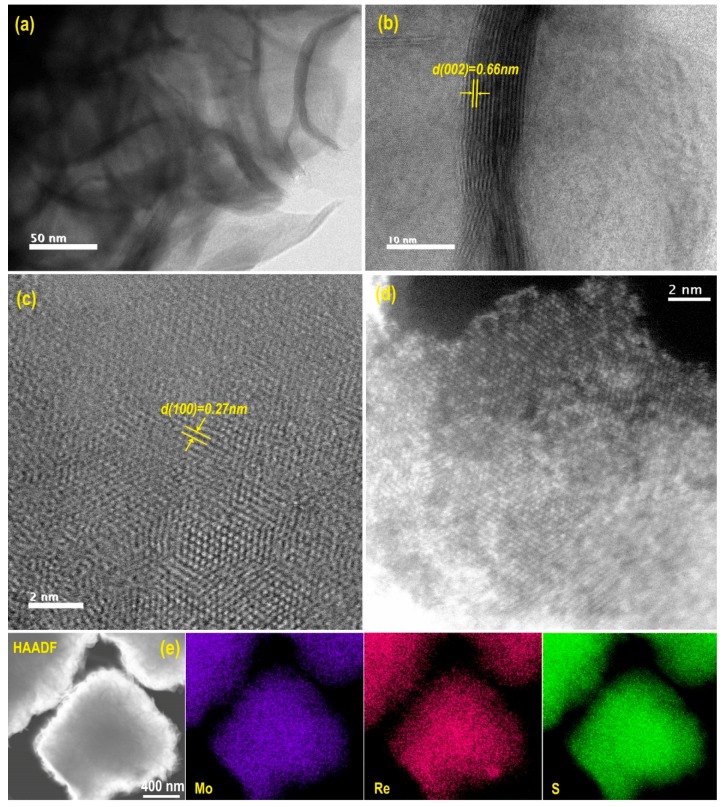
High-resolution TEM (HRTEM) images of 14.7% Re-doped MoS_2_ particle (**a**) border of the particle, (**b**) detail of the previous image, (**c**) c-axis view of the particle, (**d**) (HAADF-STEM) image of the same sample **c**, (**e**) HAADF element mapping images of Mo, Re, and S of the Re-doped MoS_2_ particle.

High-resolution transmission electron microscopy was used to characterize the microstructure of Re-doped MoS_2_ samples. Figure 4a shows the border of a flower-like particle of the 14.7% Re-doped MoS_2_ sample. As can be seen, it confirms that the flower-like particles are composed of an agglomeration of few MoS_2_ layers, whereas Figure 4b shows the detail of a few MoS_2_ stacked layers in the same sample, which are composed of about 10 atomic layers. Further, a layer spacing of 0.66 nm can be observed, corresponding to the (002) crystalline plane of 2H-MoS_2_ [21]. The most noticeable difference between the pristine MoS_2_ and Re-doped MoS_2_ samples is the curvature associated in the latter (Figure S1). Figure 4c shows a c-axis view of the same sample (14.7% Re-MoS_2_). A d-spacing of 0.27 nm is evident, which can be assigned to the (100) plane of hexagonal MoS_2_. A high-angle annular darkfield-scanning transmission electron microscopy (HAADF-STEM) image of the same sample reveals insertion of rhenium atoms in MoS_2_ layers (Figure 4d), and its homogeneous distribution over the structure is corroborated by elemental mapping (Figure 4e).

#### 2.1.5. X-Ray Photoelectron Spectroscopy

**Figure 5:**
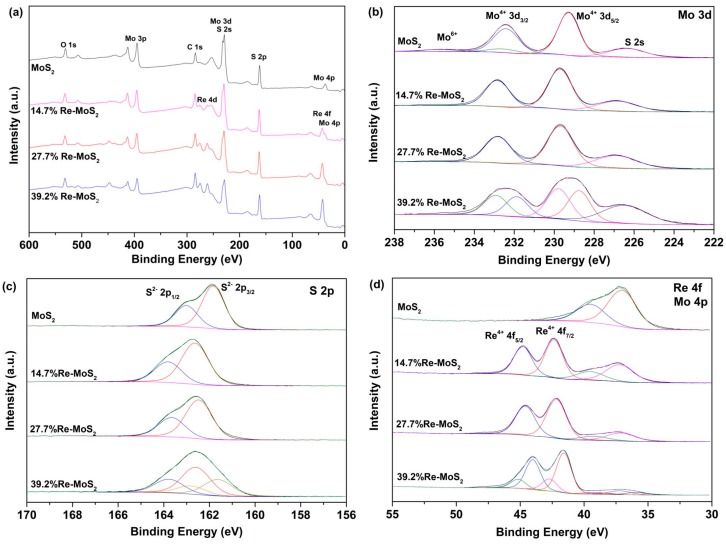
X-ray photoelectron spectra (XPS) spectra of Re-doped MoS_2_ and pristine MoS_2_. (**a**) Survey XPS of all samples, high- resolution XPS core-level of (**b**) Mo 3d, (**c**) S 2p, and states of (**d**) Re 4f and Mo 4p.

The chemical states, phase, and composition of the Re-doped MoS_2_ samples were characterized by X-ray photoelectron spectra (XPS) measurements (Figure 5). Figure 5a shows the XPS survey spectra of all samples, indicating the presence of O, Mo, C, S, and Re on the surface. Binding energies (BEs) of all peaks were calibrated on the C–C bond of C 1s at 284.5 eV (Figure S2). Figure 5b–d shows Mo 3d, S 2p, and Re 4f high-resolution spectra of pristine MoS_2_, and of 14.7%, 29.7%, and 39.2% Re-doped MoS_2_ samples. A typical doublet Mo 3d_5/2_ and Mo 3d_3/2_ with binding energies in agreement for the presence of Mo^4+^ in MoS_2_ is shown in Figure 5b [27]. Samples 14.7% Re-doped MoS_2_ and 29.7% Re-doped MoS_2_ show an upshift of 0.5 eV in comparison to the pristine MoS_2_, with peaks located at approximately 229.7 and 232.8 eV for the Mo 3d_5/2_ and Mo 3d_3/2_, respectively, in both samples. The binding energy upshift for the same samples is also observed in the S 2p region (Figure 5c). These results, together with those of HAADF-STEM analysis, confirm a substitutional n-type Re doping on the MoS_2_ structure and are consistent with previously synthesized Re-doped MoS_2_ materials [33]. The sample with the highest amount of rhenium (39.2% Re-MoS_2_) shows the most broadened spectra, having an additional low-energy component obtained by the deconvolution of the Mo 3d and S 2p spectra (Figure 5b,c), which suggests the presence of two kinds of molybdenum species [19]. This component at low binding energy can be considered as a contribution of structural defects or due to disordered structures close to the metastable 1T-MoS_2_ configuration among a 2H-MoS_2_ phase [19]. Figure 5d shows the Re 4f_7/2_ and 4f_5/2_ binding energy peaks for the 14.7%, 29.7%, and 39.2% Re-doped MoS_2_ samples, which confirms the presence of Re^4+^ in all the Re-doped samples [30]. This points out that rhenium atoms are immobilized as Re^4+^ (as rhenium sulfide) in the MoS_2_ structure. The binding energies and element analysis results of the samples are summarized in Table 1.

### 2.2. Hydrogen Evolution Reaction (HER) Performance of Pristine MoS_2_ and Re-doped MoS_2_ Samples

**Figure 6:**
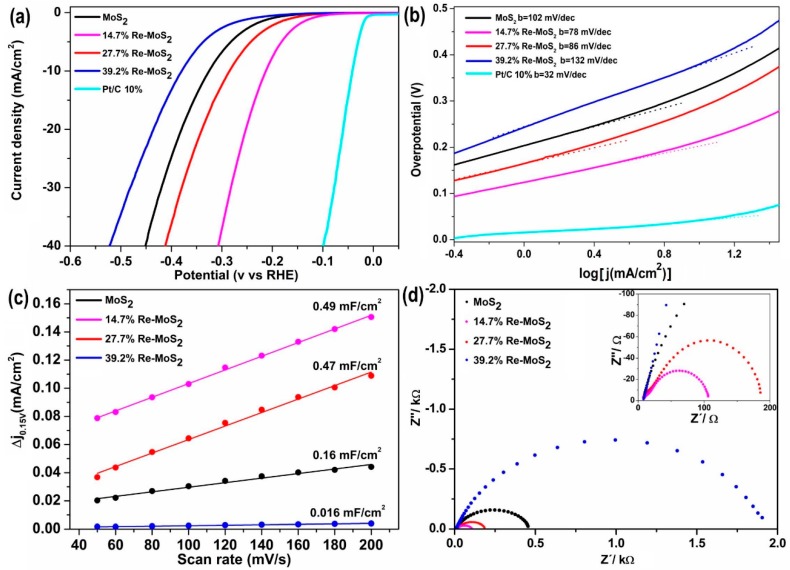
Electrocatalytic performance of Re-doped MoS_2_ and pristine MoS_2_. (**a**) Linear sweep voltammetry (LSV) curves, (**b**) Tafel plots, (**c**) electrochemical double layer capacitance (C_dl_), and (**d**) electrochemical impedance spectroscopy (EIS) plots.

HER catalytic activity for the pristine MoS_2_ and the Re-doped MoS_2_ samples is shown in Figure 6. As depicted in the electrochemical linear sweep voltammetry (LSV) in Figure 6a, the pristine MoS_2_ sample exhibits an overpotential of 326 mV at a current density of 10 mA/cm^2^ in agreement with similar MoS_2_ materials reported in the literature [34]. Although doping by rhenium clearly improves the catalytic performance of MoS_2_, it is the sample with the lowest rhenium content (14.7% Re-doped MoS_2_) that shows the highest activity among all samples towards HER, with a small overpotential of 210 mV observed at a current density of 10 mA/cm^2^. Considering the similar crystallinity and nanosheet arrangement between the pristine MoS_2_ and 14.7% Re-doped MoS_2_ sample, the superior catalytic activity of the doped sample must necessarily arise from doping, rather than from textural effects of rhenium over morphology. A decay in HER performance for samples with higher Re-doping content (39.2% Re-MoS_2_ and 27.7% Re-MoS_2_) is also evident, and this trend has been previously observed for transition metal doping in MoS_2_ materials [16,35]. Thus, the Re atoms do not function as active sites; rather, they activate the MoS_2_ basal planes. Tafel plots of all samples were derived from LSV curves to characterize their intrinsic reaction kinetics (Figure 6b). As can be seen, 14.7% Re-doped MoS_2_ exhibits the smallest Tafel slope (78 mVdec^−1^), indicating a faster reaction rate in comparison with that of the pristine MoS_2_ sample (102 mVdec^−1^). Re atomic doping increases the Tafel slope of samples, as summarized in Table 2. These results indicate that HER of 14.7% Re-doped MoS_2_ sample proceeds via a Volmer–Heyrovsky mechanism, where a proton fast discharge is followed by a rate-limiting electrochemical desorption step [36]. Figure 6c shows electrochemical double-layer capacitance (C_dl_) measured from cyclic voltammograms of Figure S3, considering its linear proportional relationship with the electrochemical active surface area (ECSA). As expected, 14.7% Re-doped MoS_2_ shows the highest C_dl_ of all samples, being approximately three times higher than that of the pristine MoS_2_ sample, suggesting a greater availability of active sites in Re-doped MoS_2_ sample. Higher ECSA in Re-doped MoS_2_ samples can be explained by the increase of catalytically active sites in basal planes, in agreement with previous results obtained in similar Re-doped MoS_2_ materials [16]. Electrochemical impedance spectroscopic (EIS) measurements were conducted to elucidate the electrode kinetics upon HER. The observed diameters of semicircles in Nyquist plots (Figure 6d) correlate with the charge transfer resistance (R_ct_) at the solid–liquid interphase. As can be seen in Figure 6d, the sample with minor rhenium content (14.7% Re-MoS_2_) displayed lower impedance than that of the pristine MoS_2_ sample. This demonstrates that low Re-doping on MoS_2_ decreases charge-transfer resistance in this material and enhances its catalytic activity in HER. The stability of the 39.2% Re-doped MoS_2_ sample was investigated by a continuous cyclic voltammetry (CV). A similar polarization curve after 1000 cycles was observed in comparison to the initial curve in Figure S4, indicating the long-term stability of 39.2% Re-MoS_2_ sample, with only slight activity degradation at the end of the cycling.

Experimental results indicate formation of 2H MoS_2_ phase with Re substitutional n-type doping, where Re doping allows tuning of morphological, structural, and electronic properties of MoS_2_ during hydrothermal synthesis. Although it has been found that hydrothermal synthesis of MoS_2_ doped with Re induces 2H-1T phase transformation [18], we cannot specifically identify this phase transformation in our results due to special features of MoS_2_ synthesized under solvothermal conditions. These include easy oxidation of samples in environmental conditions during Raman acquisition (Figure S5) [31]; difficulty in identifying the 1T/2H phase of few-layered MoS_2_ materials in TEM [37]; and existence of molybdenum polysulfides (considering solvothermal synthesis conditions) for XPS [38]. Our electrocatalytic results show that low Re doping improves overall HER catalytic activity of MoS_2_ due to the creation of new catalytically active sites in basal planes, and by decreasing charge transfer resistance in the doped material. This result agrees with n-type Re doping in MoS_2_, where the presence of extra states close to the Fermi energy is correlated with an increase of HER activity and with longer Mo-S bond length [16]. The catalytic active sites in our hydrothermal Re-doped MoS_2_ samples should correspond to activated sulfur atoms (Re-S^*^-Mo) in the basal plane of MoS_2_ [15,35]. This can explain the lower HER performance of higher rhenium content samples, where there is a decrease of Re–S^*^–Mo by formation of Re–S bonding.

## 3. Materials and Methods

### 3.1. Chemicals

All chemical reagents used in the experiments were obtained from commercial sources as guaranteed grade reagents. Thiourea CH_4_N_2_S (molecular weight 76.12 g/mol, purity ≥ 99.0%), ammonium molybdate (NH_4_)_2_MoO_4_ (molecular weight 196.01 g/mol, purity ≥ 99.98%), ammonium perrhenate NH_4_ReO_4_ (molecular weight 268.24 g/mol, purity ≥ 99.0%), and Pt/C 10% (molecular weight 195.08, purity 9.8–10.2%) were purchased from Sigma-Aldrich. All chemical reagents were of analytical grade and utilized without any further purification.

### 3.2. Synthesis of Re-Doped MoS_2_ and Pristine MoS_2_

The synthesis of Re-doped MoS_2_ consists in the hydrothermal treatment of 1.0 to 2.0 mmol (NH_4_)_2_MoO_4_, 6.0 mmol CH_4_N_2_S, and 0.2 to 1.0 mmol NH_4_ReO_4_ (Table 3). This mixture was dissolved in 18 mL of deionized water, placed into a Teflon-lined 20 mL stainless steel autoclave, and heated in an electric oven for 24 h at 180 °C. The obtained product, a black powder, was washed several times with ethanol and dried in vacuum overnight. The as-prepared samples were annealed by heating at 10 °C per min rate in a conventional tube furnace under Ar flow (20 sccm) up to 400 °C for 2.0 h. The same procedure was used to prepare pristine MoS_2_ as the control experiment, but without adding NH_4_ReO_4_.

### 3.3. Characterization Techniques

X-ray diffraction (XRD) measurements of the samples were gathered in a Bruker diffractometer model D8 (Bruker, Billerica, USA) using the Cu Kα radiation (40 kV, 30 mA) with a wavelength of 0.154 nm. Crystalline phases were identified using standard JCPDS files. Raman spectroscopy measurements were collected at room temperature. Samples were measured using a confocal WITec alpha300 system instrument (WITec, Ulm, Germany) equipped with a 100× objective and 300 lines/mm grating. Measurements were performed using a green (532 nm) laser excitation wavelength. The Si Raman band at 520 cm^−1^ was used as a reference for the calibration of the Raman shift. Field-emission scanning electron microscopy (SEM) micrographs were obtained in an SEM LEO 1420VP, Oxford Instruments, equipped with an energy-dispersive X-ray spectroscopy (EDS) system (Oxford Instruments, Oxford, UK). Transmission electron microscopy (TEM) in STEM mode was conducted using a JEOL 2000FS (JEOL, Peabody, MA, USA) operating at 200 kV. The analysis of images was carried out using the Digital Micrograph Gatan™ software. X-ray photoelectron spectra (XPS) of both catalysts were carried out in a SPECS GmbH custom-made system using a PHOIBOS 150 WAL hemispherical analyzer and a μ-FOCUS 500 X-ray source (SPECS, Berlin, Germany). All data were acquired using monochromated Al Kα X-rays (1486.6 eV, 110 W), a pass energy of 50 eV, and high-intensity lens mode. The charge referencing was done against adventitious carbon (C 1s 284.5 eV). Spectra were presented without smoothing, and a Shirley-type background was subtracted. Fits of the experimental peaks were obtained using combinations of Gaussian/Lorentzian lines with a 70/30 proportion using CasaXPS from Casa Software Ltd. The effective atomic concentrations were corrected according to sensitivity factors.

### 3.4. Electrochemical Measurements

Electrochemical measurements were obtained with a computer-controlled Zahner IM6ex, in a standard three-electrode cell using an Ag/AgCl (in 1.0 n KCl solution) electrode as the reference electrode, a platinum wire as the counter electrode, and glassy carbon (GC) electrodes carrying the catalyst as working electrodes. The working electrode was fabricated as follows: 4 mg of catalyst and 80 μL of 5 *wt*% Nafion solution were dispersed in 1 mL of a solution of deionized water and ethanol (3:1 in volume ratio). After stirring by ultrasonication for 1 h, 5μL of the resulting ink was drop-casted onto the top of a glassy carbon electrode with a 3 mm diameter. The catalyst-coated GC electrode was dried at 80 °C for 2 h to yield a catalyst loading of 0.285 mg cm^−2^. Linear sweep voltammetry (LSV) with a scan rate of 2 mV s^−1^ was conducted in 0.5 M H_2_SO_4_ (purged with Ar), without applying iR correction. To determine double layer capacitance (C_dl_) values, cyclic voltammograms (CVs) were collected at different scan rates (50, 60, 80, 100, 120, 140, 160, 180, and 200 mV s^−1^) in the range of potential from 0.1 to 0.2 V vs. RHE. Electrochemical impedance spectroscopy (EIS) analyses were performed at an overpotential of 200 mV (vs RHE) from 100 kHz to 0.1 Hz with the amplitude fixed at 5 mV in the same configuration. All catalysts were electrochemically cleaned by cyclic voltammetry from OPC to −0.55 V for 20 cycles at a scan rate of 10 mVs^−1^ prior to measurements.

## 4. Conclusions

In conclusion, hydrothermally synthesized Re-doped MoS_2_ materials were characterized and investigated as unsupported catalyst for HER. Re doping shows a homogeneous distribution within the catalyst structure and maintained 2H-MoS_2_ crystallographic phase, with no indication of minority phases. Optimization of Re-doping on the MoS_2_ structure enables improving its catalytic performance through activation of its basal plane for HER and by decreasing charge transfer resistance of the doped material. This result agrees with the presence of extra states close to Fermi level for n-type doping. Rhenium doping on MoS_2_ reduces overpotential from 326 to 210 mV at 10 mA cm^−2^ for sample with 14.7% Re.

## Figures and Tables

**Table 1:** Binding energies (eV) of core electrons of the pristine MoS_2_ and Re-doped MoS_2_ samples.

Sample	Mo 3d_5/2_	S 2p_3/2_	Re 4f_7/2_	Composition
Pristine MoS_2_	229.3	161.8	-	MoS_1.64_
14.7% Re-doped MoS_2_	229.7	162.6	42.4	Mo_0.85_Re_0.15_S_1.76_
27.7% Re-doped MoS_2_	229.7	162.5	42.2	Mo_0.72_Re_0.28_S_1.73_
39.2 % Re-doped MoS_2_	229.8, 228.8	162.8, 161.7	42.7, 41.6	Mo_0.61_Re_0.39_S_1.83_

**Table 2:** Summary of electrochemical measurements of pristine MoS_2_ and Re-doped MoS_2_ samples.

Sample	Onset Potential (mV)	η10 (mV)	Tafel Slope (mVdec^−1^)	Rct (Ωcm^2^)
Pristine MoS_2_	203	326	102	32.58
14.7% Re-doped MoS_2_	123	210	78	7.77
27.7% Re-doped MoS_2_	164	285	97	17.45
39.2% Re-doped MoS_2_	244	379	132	157.86
Pt/C 10%	20	42	32	-

**Table 3:** Synthesis parameters for pristine MoS_2_ and Re-doped MoS_2_ samples.

Sample	(NH_4_)_2_MoO_4_	(NH_4_)ReO_4_	CH_4_N_2_S
Pristine MoS_2_	2.0 mmol	-	6.0 mmol
14.7% Re-doped MoS_2_	1.8 mmol	0.2 mmol	6.0 mmol
27.7% Re-doped MoS_2_	1.5 mmol	0.5 mmol	6.0 mmol
39.2% Re-doped MoS_2_	1.0 mmol	1.0 mmol	6.0 mmol

## Supplementary Materials

The following are available online at https://www.mdpi.com/1420-3049/24/24/4631/s1, Figure S1: TEM images of pristine MoS_2_ particle (a), and 39.2% Re-doped MoS_2_ particle (b); Figure S2: High-resolution spectra of C 1s of Re-MoS_2_ composites with different loadings of rhenium; Figure S3: Cyclic voltammograms of pristine MoS_2_ (a), 14.7% Re-doped MoS_2_ (b), 27.7% Re-doped MoS_2_ (c), and 39.2% Re-doped MoS_2_ (d) samples; Figure S4: Polarization curves of 39.2% Re-doped MoS_2_ sample before and after 1.000 cycles; Figure S5: Oxidation of pristine MoS_2_ sample in environmental conditions during Raman acquisition; Table S1: Atomic ratios of the samples derived from peak deconvolution of XPS spectra.
