# Supplementary material for: Electrochemical Hydrogen Evolution over Hydrothermally Synthesized Re-Doped MoS2 Flower-Like Microspheres
Source: Molecules. 2019 Dec 17;24(24):4631. doi: 10.3390/molecules24244631 (PMC6943669; doi:10.3390/molecules24244631)
Supplement: Supplementary file 1 [file molecules-24-04631-s001.pdf]

Supplementary Materials

Electrochemical Hydrogen Evolution over Hydrothermally Synthesized Re-doped MoS_2 Flower-like Microspheres

Juan Aliaga[1*], Pablo Vera[1], Juan Araya[2], Luis Ballesteros[3], Julio Urzúa[4], Mario Farías[5], Francisco Paraguay-Delgado[6], Gabriel Alonso-Núñez[5], Guillermo González[7], Eglantina Benavente[1*]

1 Departamento de Química, Universidad Tecnológica Metropolitana, Las Palmeras 3360, Ñuñoa, Santiago, Chile; p.vera1@gmail.com
2 Centro de Investigaciones Costeras de la Universidad de Atacama, Universidad de Atacama, Copayapu 485, Copiapó, Chile; juan.araya@uda.cl
3 Instituto de Ciencias Químicas Aplicadas, Universidad Autónoma de Chile, El Llano Subercaseaux 2801, San Miguel, Chile; luis.ballesteros@uautonoma.cl
4 Departamento de Ciencias Farmacéuticas, Facultad de Ciencias, Universidad Católica del Norte, Casilla 1280, Antofagasta, Chile; j.urzua,ahumada@gmail.com
5 Centro de Nanociencia y Nanotecnología, Universidad Nacional Autónoma de México, EnsenadaC. P. 22860, Mexico; Mario@cnyn.unam.mx (M.F.); galonso@cnyn.unam.mx (G.A.-N.)
6 Departamento de Física de Materiales, Centro de Investigación Materiales Avanzados S.C., Miguel de Cervantes 120, CP 31136, Chihuahua, México; francisco.paraguay@cimav.edu.mx
7 Departamento de Química, Facultad de Ciencias, Universidad de Chile. Las Palmeras 3425, Santiago, Chile; ggonzale@uchile.cl

Contents

Fig. S1 TEM images of pristine MoS_2 particle (a), and 39.2% Re-doped MoS_2 particle (b).

Fig S2. High-resolution spectra of C 1s (b) of Re-MoS_2 composites with different loadings of rhenium.

Fig S3. Cyclic voltammograms of pristine MoS_2 (a), 14.7% Re-doped MoS_2 (b), 27.7% Re-doped MoS_2 (c), and 39.2% Re-doped MoS_2(d) samples.

Fig S4. Polarization curves of 39.2% Re-doped MoS_2 sample before and after 1.000 cycles.

Fig S5. Oxidation of pristine MoS_2 sample in environmental conditions during Raman acquisition

Table S1. Atomic ratios of the samples derived from peak deconvolution of XPS spectra.

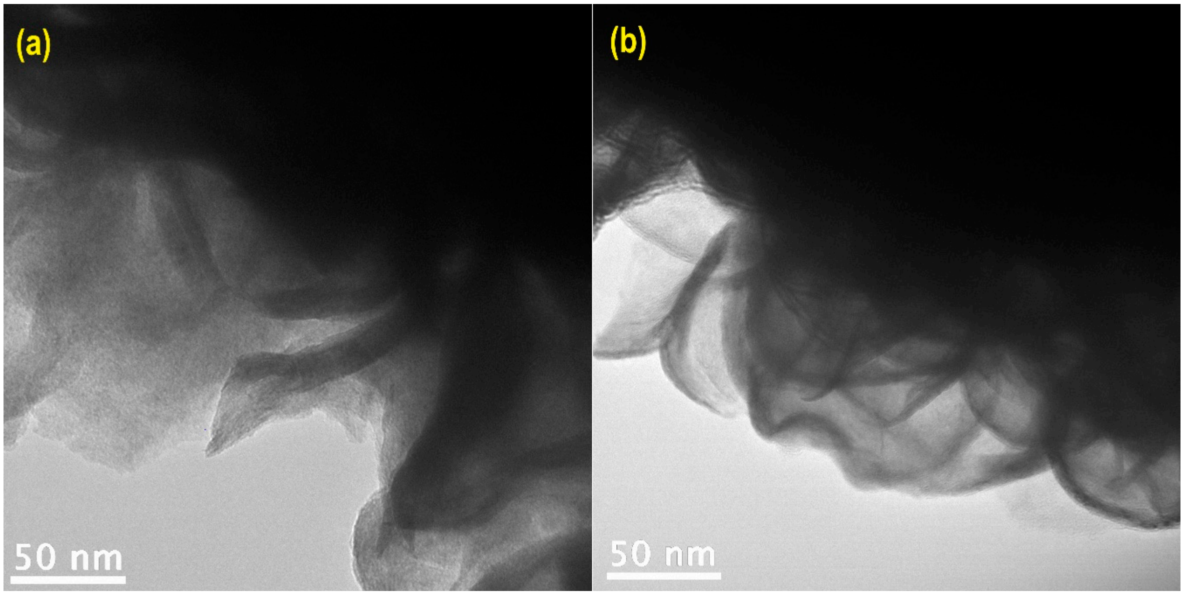

Figure. S1 TEM images of pristine MoS_2 particle (a), and 39.2% Re-doped MoS_2 particle (b).

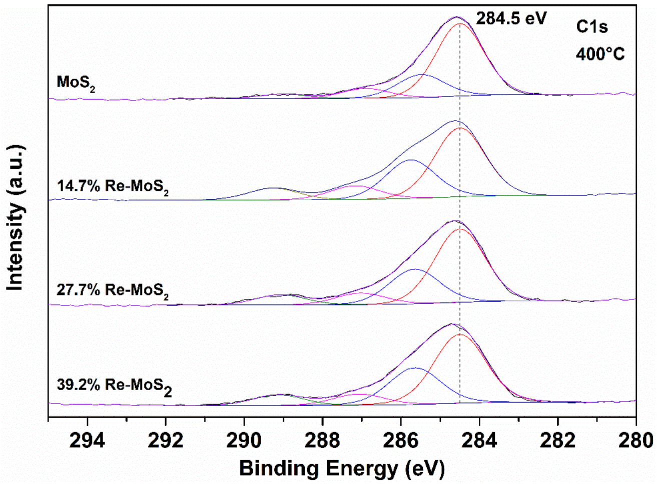

Figure S2. High-resolution spectra of C 1s (b) of Re-doped MoS_2 composites with different loadings of rhenium.

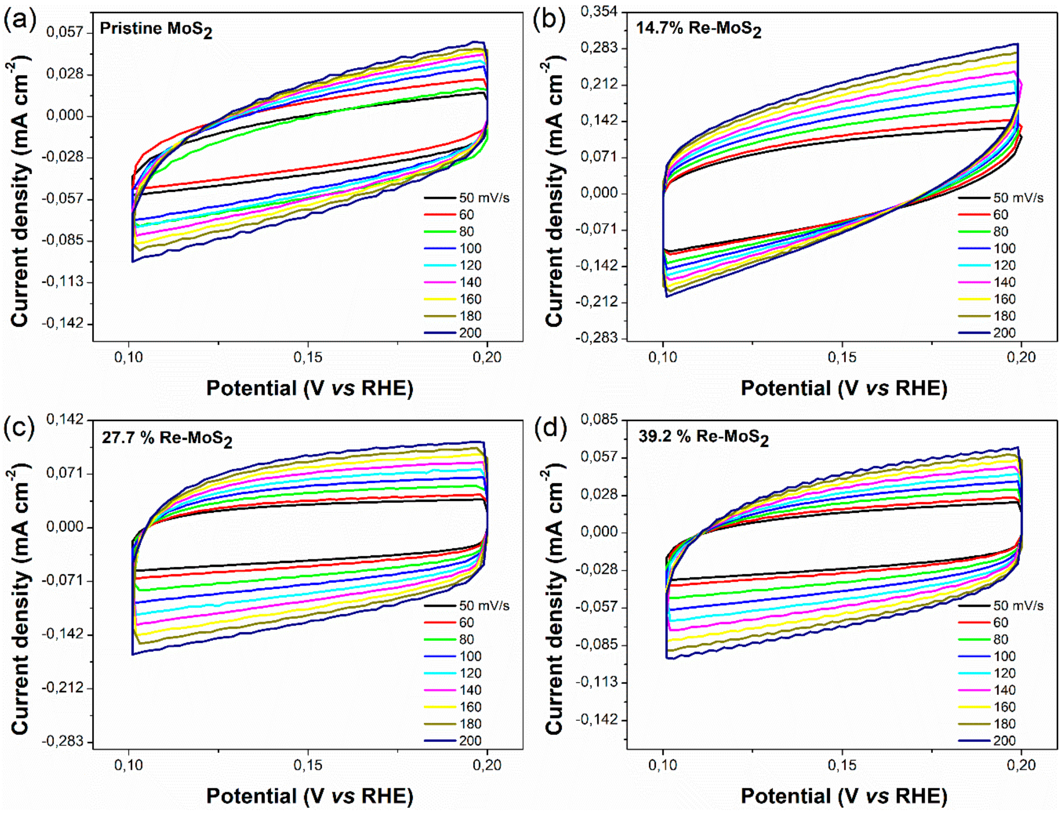

Figure S3. Cyclic voltammograms of pristine MoS_2 (a), 14.7% Re-doped MoS_2 (b), 27.7% Re-doped MoS_2 (c), and 39.2% Re-doped MoS_2(d) samples.

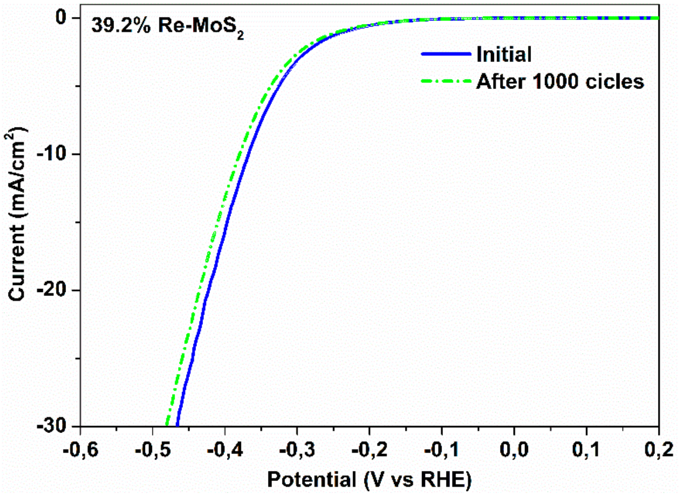

Figure S4. Polarization curves of 39.2% Re-doped MoS_2 sample before and after 1.000 cycles.

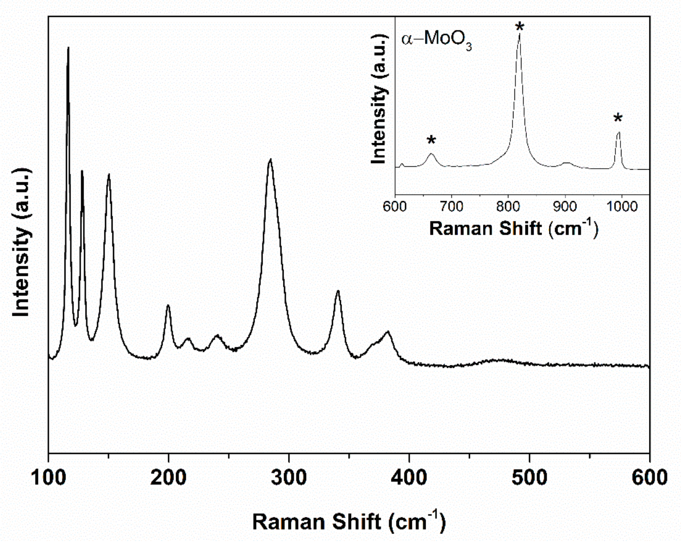

Figure S5. Oxidation of pristine MoS_2 sample in environmental conditions during Raman spectrum acquisition

Table S1. Atomic ratios of the samples derived from peak deconvolution of XPS spectra.

Sample	at.% Molybdenum	at.% Rhenium	S/Mo+Re	Re/Re+Mo
Pristine MoS_2	100	0	1.64	0
14.7 % Re-doped MoS_2	85.3	14.7	1.76	0.147
27.7 % Re-doped MoS_2	72.3	27.7	1.73	0.277
39.2 % Re-doped MoS_2	60.8	39.2	1.83	0.392
